# Optogenetic neuronal stimulation of the lateral cerebellar nucleus promotes persistent functional recovery after stroke

**DOI:** 10.1038/srep46612

**Published:** 2017-06-01

**Authors:** Aatman M. Shah, Shunsuke Ishizaka, Michelle Y. Cheng, Eric H. Wang, Alex R. Bautista, Sabrina Levy, Daniel Smerin, Guohua Sun, Gary K. Steinberg

**Affiliations:** 1 Department of Neurosurgery and Stanford Stroke Center, Stanford University School of Medicine, Stanford, CA 94305, USA.

## Abstract

Stroke induces network-wide changes in the brain, affecting the excitability in both nearby and remotely connected regions. Brain stimulation is a promising neurorestorative technique that has been shown to improve stroke recovery by altering neuronal activity of the target area. However, it is unclear whether the beneficial effect of stimulation is a result of neuronal or non-neuronal activation, as existing stimulation techniques nonspecifically activate/inhibit all cell types (neurons, glia, endothelial cells, oligodendrocytes) in the stimulated area. Furthermore, which brain circuit is efficacious for brain stimulation is unknown. Here we use the optogenetics approach to selectively stimulate neurons in the lateral cerebellar nucleus (LCN), a deep cerebellar nucleus that sends major excitatory output to multiple motor and sensory areas in the forebrain. Repeated LCN stimulations resulted in a robust and persistent recovery on the rotating beam test, even after cessation of stimulations for 2 weeks. Furthermore, western blot analysis demonstrated that LCN stimulations significantly increased the axonal growth protein GAP43 in the ipsilesional somatosensory cortex. Our results demonstrate that pan-neuronal stimulations of the LCN is sufficient to promote robust and persistent recovery after stroke, and thus is a promising target for brain stimulation.

Stroke is a leading cause of death and disability in the US, yet treatment options are very limited. Functional recovery can occur after stroke and is attributed in part to rewiring of neural connections in areas adjacent or remotely connected to the infarct^1^,^2^,^3^,^4^. Multiple strategies have been used to enhance recovery, including pharmacological treatment, rehabilitation, cell transplantation and brain stimulation^5^,^6^,^7^,^8^,^9^,^10^,^11^. In particular, brain stimulation is a promising neurorestorative technique as it allows direct manipulation of the target area’s excitability^11^,^12^,^13^. Enhancing cortical excitability through electrical stimulation, transcranial direct current stimulation, or transcranial magnetic stimulation after stroke has been linked to improved recovery in animal and human studies of stroke^11^,^14^,^15^. However, it is unclear whether the beneficial effect of stimulation is due to activation of neuronal or non-neuronal cell types, as current brain stimulation techniques nonspecifically activate or inhibit all cell types in the target area (neurons, glia, endothelial cells, oligodendrocytes)^16^,^17^,^18^. To overcome this, we use the optogenetics approach to selectively stimulate neurons in the brain and address its involvement in stroke recovery. Optogenetics can manipulate specific cell types and circuits with high spatiotemporal precision^19^,^20^, thus is an ideal technique to dissect the underling cell types driving recovery^21^,^22^.

Previously we demonstrated that optogenetic stimulations of layer V neurons in the ipsilesional primary motor cortex (iM1) can promote stroke recovery^23^. Although these iM1-stimulated mice exhibited significant improvement in functional recovery, their performance in the rotating beam test only returned to ~50% of the pre-stroke baseline^23^, suggesting the possibility of even further improvement. In an effort to optimize our stimulation target to further enhance stroke recovery, we investigated the deep cerebellar nuclei, specifically the LCN, because it sends major motor output to the cerebral cortex^24^,^25^. The LCN is the largest and most lateral nucleus of the four deep cerebellar nuclei. It sends major excitatory output to the cortex via the dentato-thalamo-cortical pathway, including motor, premotor, somatosensory and non-motor areas that are involved in functions such as balance, coordination, movement planning and visuospatial function^26^,^27^,^28^ (Fig. 1). Previous studies have demonstrated that lesioning of the dentato-thalamo-cortical pathway reduced excitability in the contralateral cortex, while stimulations of the dentato-thalamo-cortical pathway enhanced contralateral cortical excitability^29^. Using electrical stimulation, studies have shown that chronic electrical stimulation in the LCN after stroke can enhance stroke recovery^30^,^31^. However, it is unclear whether the stimulation effect is due to direct neuronal activation, and whether the pro-recovery effect is persistent. In this study we used optogenetics to selectively stimulate only neurons of the contralesional LCN (cLCN) after stroke and examined its effects on functional recovery. We also addressed whether the effects of cLCN stimulation are transient or persistent. Furthermore, we investigated the expression of the axonal growth protein GAP43, a key growth cone phosphorylation protein that has been highly linked to neurite outgrowth and plasticity^32^,^33^,^34^. As increasing neuronal activity leads to activity-dependent processes such as axonal sprouting, we hypothesized that LCN stimulations would have a positive effect on GAP43 expression.

## Results

### Validation of the cLCN location

**Figure 2:**
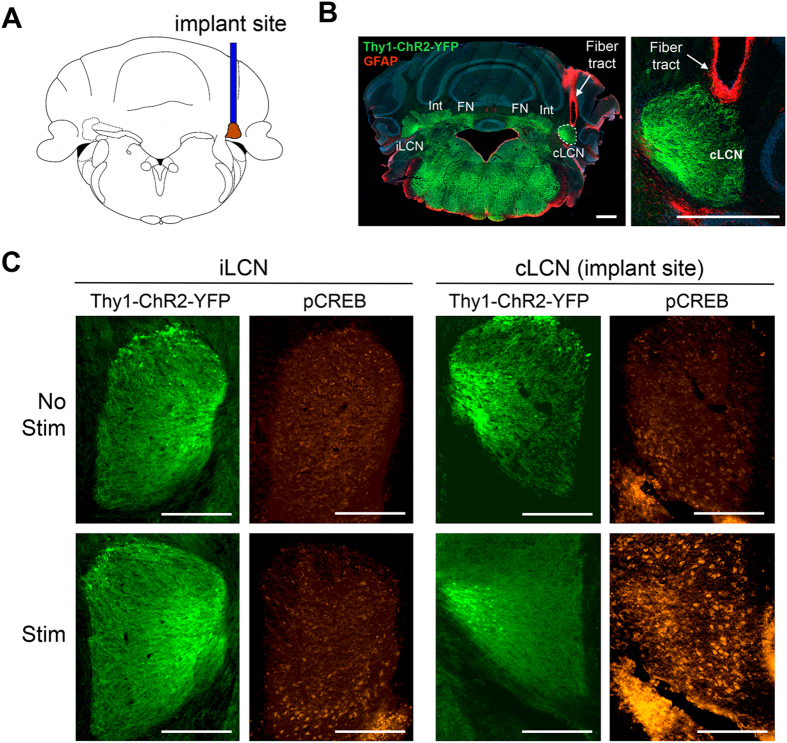
Neuronal stimulation of the contralesional cerebellar dentate nucleus (cLCN) activates pCREB expression. (**A**) Diagram shows the location of implant site in the cLCN. (**B**) Representative image of cLCN implant location in the Thy1-ChR2-YFP mice. Positive expression of ChR2-YFP was observed in the cLCN (green) and GFAP immunostaining (red) illustrate fiber tract to the cLCN. Int = interposed nuclei, FN = fastigial nuclei. The area surrounding the LCN is the white matter. Scale Bar = 500 um. (**C**) Representative images of pCREB expression (red) and corresponding Thy1-ChR2-YFP expression (green) in the ipsilesional LCN (iLCN) and contralesional LCN (cLCN) in cLCN-stimulated and non-stimulated mice. Note that selective pCREB expression in the cLCN was observed in cLCN-stimulated mice at 90 minutes after stimulation, indicating that cLCN-stimulations led to neuronal activation in the cLCN. Scale bar = 250 um.

To investigate the effects of neuronal stimulations in the cLCN, we used the transgenic mouse line 18 that expresses channelrhodopsin fused to yellow fluorescent protein under the Thy1 pan-neuronal promoter (Thy1-ChR2-YFP) (Fig. 2B). ChR2 is a membrane bound protein that is expressed mostly in axons and dendrites^35^. All mice had an optical fiber stereotaxically implanted into the right cLCN (Fig. 2A). The mouse cerebellar dentate nucleus is anatomically very small, thus we used several methods to verify that we had successfully targeted the cLCN. Since stimulation of cLCN is expected to activate the dentato-thalamo-cortical pathway and innervate motor movements, we first examined whether cLCN stimulation could evoke movements in the affected forelimb and whiskers. We tested 3 coordinates: cLCN coordinate, medial off-target coordinate (medial to the cLCN) and lateral off-target coordinate (lateral to the cLCN) (see Methods for details on coordinates). As expected, stimulations in the cLCN elicited reliable movements in the affected forelimb (same side as the implant) during the stimulation period. Some whisker movements were detected as well (See Supplementary Video 1 for the visual validation of cLCN stimulation-induced forelimb movements. Both medial and lateral off-target coordinates did not elicit movements in the affected forelimb, although some whisker movements were noted (Supplementary Video 2 and 3).

To perform histological validation of the fiber implant location, brain sections were immunostained with glial fibrillary acidic protein (GFAP), a marker that targets reactive astrocytes around the fiber tract. Correct placement of the fiber in the cLCN was validated by GFAP-positive cells along the fiber tract and ends at the tip of the cLCN (Fig. 2B). We had expected that during stimulations the laser from the fiber tip would illuminate the cLCN. To test this, we used a photothrombotic method which can lesion a target area through photo-activation of an injected light-sensitive dye (Rose Bengal)^36^. Using this photothrombotic method, we demonstrated that cLCN was successfully and completely lesioned in the cLCN coordinate mice (Supplementary Fig. 1a). The medial off-target coordinate did not lesion the cLCN (Supplementary Fig. 1b) and the lateral off-target coordinate partially lesioned the cLCN (Supplementary Fig. 1c). The partial cLCN lesion in the lateral off-target coordinate may be due to the higher laser power (2 mW) used in the photothrombotic method which would result in a broader lesioning effect than low power, such as the power used in our stimulation paradigm (0.2–0.4 mW).

### Stimulation of cLCN activates pCREB

**Figure 3:**
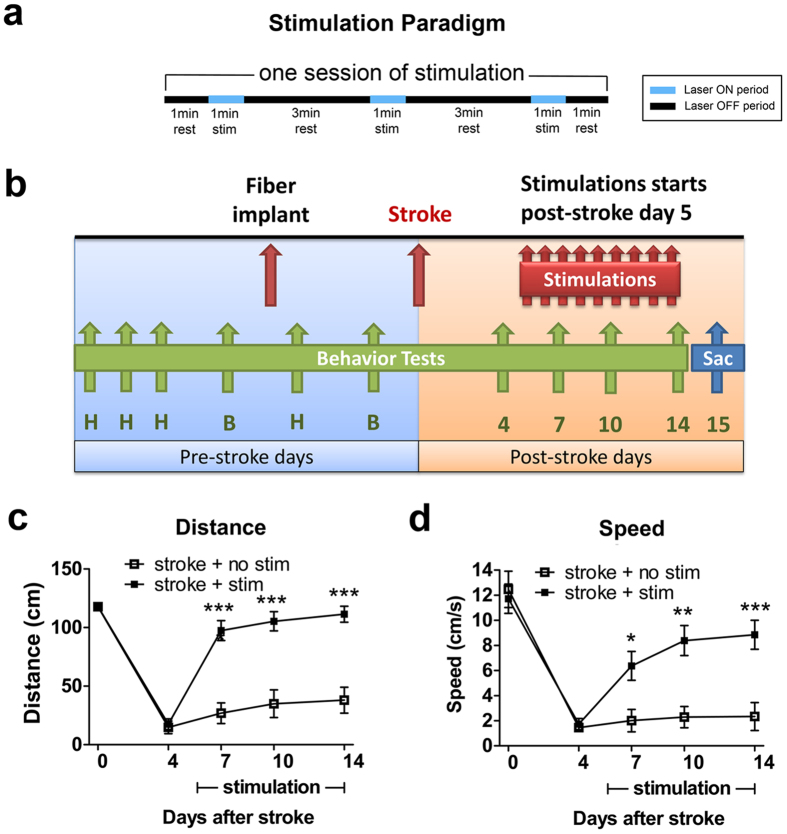
Repeated neuronal stimulations of the cLCN promote post-stroke recovery. (**a**) Diagram illustrates the stimulation paradigm used. One session of stimulation consists of three 1-minute stimulations with 3-minute rest periods in between^23^. Laser ON periods (blue) and laser OFF periods (black) are indicated on the diagram. (**b**) Experimental design: Mice were pre-trained (H) on the rotating beam test prior to recording the pre-implant baseline (B) and pre-stroke baseline data (Day 0). Each mouse received one session of stimulation daily, from post-stroke Day 5 and continued until Day14. Behavior tests were performed at post-stroke Days 4, 7, 10 and 14. Mice were sacrificed at Day 15 for histological and western blot analysis. cLCN-stimulated stroke mice exhibit significant improvement as early as Day 7 after stroke, as reflected by their performance in both distance traveled (**c**) and speed (**d**). n = 15 for stroke + no stim, n = 18 for stroke + stim. Two-way ANOVA with Bonferroni’s post hoc test, *P < 0.05, **P < 0.01,***P < 0.001. Data are expressed as mean ± s.e.m.

To examine whether stimulation of cLCN leads to neuronal activation, we used pCREB as an activation marker, as it has been previously demonstrated to be a stable readout of changes in neuronal activity after optogenetic stimulation^37^. Naïve Thy1-ChR2-YFP mice with cLCN implants underwent one session of stimulation, which consisted of three 1-minute stimulations (Fig. 3a). At 90 minutes after one session of cLCN stimulation, we observed increased pCREB expression in the cLCN (Fig. 2C) of stimulated mice, and this was absent in the non-stimulated mice. In the ipsilesional LCN (iLCN), we only observe low or non-detectable levels of pCREB in both non-stimulated and stimulated mice (Fig. 2C). During cLCN stimulation, we also observed reliable forelimb and whisker movements (same side as the implant) during stimulation (Supplementary Video 1), indicating successful activation of the dentato-thalamo-cortical pathway.

### Repeated cLCN stimulations promotes functional recovery after stroke

To test whether repeated cLCN stimulations after stroke can enhance recovery, we used the same stimulation paradigm and experimental design as previously reported for iM1 stimulation^23^ (Fig. 3a,b). Each mouse received one session of stimulations daily (each session consists of three 1-minute stimulations, 10 hz 20 ms 473 nm) (Fig. 3a) from post-stroke Days 5–14 (Fig. 3b). Functional recovery was evaluated using the rotating beam test, a motor-sensory test that detects neurological deficit and involves motor coordination and balance. Animals were trained on the rotating beam test during the pre-stroke days, and pre-stroke baseline was collected (Day 0). After stroke, the rotating beam test was performed on post-stroke Days 4, 7, 10 and 14 to evaluate recovery of motor function (Fig. 3b). After repeated cLCN stimulations, we found that cLCN-stimulated mice exhibited a significantly faster and robust recovery as early as post-stroke Day 7 in both distance traveled (Fig. 3c) and speed (Fig. 3d), when compared to non-stimulated mice. By post-stroke Day 14, cLCN-stimulated mice exhibited a similar performance in distance traveled compared to their pre-stroke baseline, and their performance in speed returned to ~80% of their pre-stroke baseline.

### Repeated cLCN stimulations increased GAP43 expression

**Figure 4:**
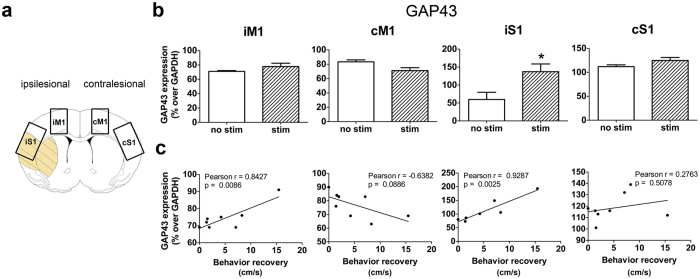
Repeated neuronal stimulations of the cLCN increased GAP43 expression in the ipsilesional cortex. (**a**) Diagram depicts the cortical regions dissected for western blot analysis: ipsilesional motor cortex (iM1), ipsilesional somatosensory cortex (iS1) and contralesional motor cortex (cS1). (**b**) Western analysis indicates that repeated cLCN stimulations after stroke significantly increased protein expression of plasticity marker GAP43 in the iS1. n = 4 for stroke + no stim and n = 4 for stroke + stim, *P < 0.05, Student’s t-test, two-tailed. Data are expressed as mean ± s.e.m. (**c**) Pearson correlation analysis indicates that GAP43 protein expression positively correlates with behavior recovery (speed) in iM1 and iS1 at post-stroke Day 14 (n = 8). Data are expressed as mean ± s.e.m.

Because increasing neuronal activity can enhance axonal growth^32^,^33^,^34^, we investigated whether cLCN stimulations affect GAP43 (growth associated protein 43) expression at Day 15 after stroke. GAP43 is a marker of axonal growth and increased GAP43 expression in the perilesional areas has been highly linked with axonal sprouting^32^,^33^,^34^. Using western blot analysis, we investigated GAP43 expression in the ipsilesional and contralesional primary motor cortex (iM1 and cM1) and somatosensory cortex (iS1 and cS1) (Fig. 4). Our results indicate that repeated cLCN-stimulated mice exhibited significantly higher GAP43 protein expression in the iS1 (Fig. 4b) (P = 0.04). Using Pearson correlation analysis, we found that GAP43 expression in the iM1 and iS1 was significantly and positively correlated with improved recovery (*P* = 0.0086 and *P* = 0.0025, respectively, Fig. 4c). This result is consistent with the literature that GAP43 expression is observed in sprouting axons and has been positively correlated with improved functional outcome^1^,^2^,^33^. Interestingly, the Pearson correlation coefficient indicated a strong trend of negative correlation between GAP43 expression and recovery in the cM1 (*P* = 0.0886), suggesting that decreasing GAP43 expression in the cM1 may play a role in recovery.

### The pro-recovery effect of repeated cLCN stimulations is persistent

**Figure 5:**
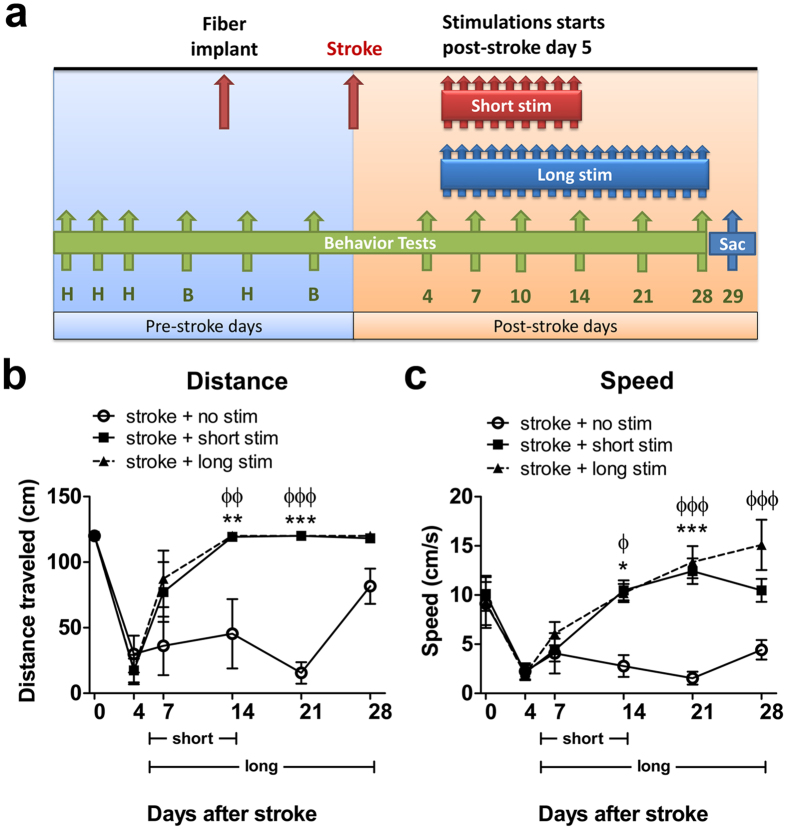
The pro-recovery effect of optogenetic cLCN stimulation is persistent. (**a**) Experimental design: Mice were pre-trained (H) on the rotating beam test prior to collecting pre-implant baseline (B) and pre-stroke baseline data (B) (Day 0). Optogenetic neuronal stimulations began at post-stroke Day 5 and continued until Day 14 (short stim group) or Day 28 (long stim group). Behavior tests were performed at post-stroke Days 4, 7, 14, 21, and 28. Mice were sacrificed at post-stroke Day 29 for histological analysis. (**b**) Both cLCN short stim and long stim stroke mice exhibited significant recovery by traveling longer distances and faster speeds (**c**). Note that the short stim group exhibit persistent recovery after Day 14 even when stimulations had stopped. Additional stimulations in the long stim group did not further enhance recovery. *P < 0.05, **P < 0.01, ***P < 0.001, Two-way ANOVA with Bonferroni’s post hoc test indicates a significant difference between the short stim and no stim groups, ^ϕ^*P* < 0.01, ^ϕϕ^*P* < 0.01, ^ϕϕϕ^*P* < 0.001 indicates a significant difference between long stim and no stim group. n = 4 for stroke + no stim, n = 5 for stroke + short stim, n = 4 for stroke + long stim. Data are expressed as mean ± s.e.m.

An important question in our study is whether the pro-recovery effect of cLCN stimulations is transient or persistent. To address this, we ran a separate cohort of animals consisting of three groups: a non-stimulated group, a short stimulated group (stimulations from post-stroke Days 5–14) and a long stimulated group (post-stroke Days 5–28). The same stimulation paradigm was used where each mouse received one session of stimulation daily (Fig. 3a); the experimental timeline is outlined in Fig. 5a. The rotating beam test was performed prior to Day 0 (baseline) and on post-stroke Days 4, 7, 14, 21, and 28 (Fig. 5a). cLCN-stimulated mice performed significantly better on the rotating beam test in distance traveled and speed by post-stroke Day 14 (Fig. 5b,c) when compared to non-stimulated stroke mice. The short stimulated group maintained their recovered state beyond post-stroke Day 14, even after the stimulations had stopped, indicating that the effect of cLCN stimulations is persistent. Interestingly, the short stimulated group exhibited a comparable outcome to the long stimulated group, suggesting that prolonged stimulation is not necessary to achieve persistent recovery.

## Discussion

The LCN is a deep nucleus within the cerebellum that sends major excitatory output to the forebrain through the dentato-thalamo-cortical pathway. It sends widespread excitatory output to multiple motor, premotor and nonmotor areas^26^,^27^,^28^. In this study we demonstrate that selective neuronal stimulations in the cLCN can promote robust recovery after stroke (Fig. 3), and more importantly, the effect of the cLCN stimulations is persistent, as stimulated mice maintained their recovery state for 2 weeks after the stimulations were stopped (Fig. 5). Furthermore, cLCN-stimulated mice exhibited increased GAP43 expression in the ipsilesional S1 and this GAP43 increase was positively correlated with recovery (Fig. 4), suggesting that cLCN stimulations may enhance structural plasticity such as axonal sprouting. Similar to our previous iM1 stimulations^23^, cLCN stimulations did not affect infarct size assessed by CD68 and GFAP immunohistochemistry (Supplementary Fig. 2). Since we started our stimulations at post-stroke Day5, it is unlikely that stimulations will affect infarct size since injury in the infarct mostly matured by 48–72 hours after stroke. Our results indicate that selective neuronal cLCN stimulation is effective and may produce long term effects on brain repair and remodeling.

Previous studies using electrical stimulation have demonstrated that chronic electrical stimulation of LCN (5–6 weeks, 12-hour stimulations per day) in rats after stroke can enhance fine motor tasks in the Montoya staircase test and pasta matrix retrieval task^30^,^31^. In addition, they reported that electrical stimulation in the LCN can enhance synaptic protein expression in the perilesional areas^31^. Although the stroke model and functional measures were different in these studies, the pro-recovery effect of LCN stimulations on functional outcome is similar to our study. For example, Cooperrider *et al*. 2014 reported significant recovery in LCN-stimulated rats starting at 2 weeks post-stroke and the pasta retrieval performance at this timepoint was already similar to their pre-stroke baseline^31^. Similarly, our cLCN-stimulated mice also exhibited significantly improved performance on the rotating beam at 2 weeks after stroke, at a level similar to the pre-stroke baseline (Figs 3 and 5). Despite similar functional outcomes, it was unclear which cell types were responsible for their stimulation’s effect on recovery and whether the effect was transient or persistent, as most electrical stimulation studies have reported transient short-term effects. In our study, we addressed this question and demonstrated that selective neuronal stimulation in the cLCN using the optogenetics approach can promote robust recovery and the stimulation effect is persistent, as cLCN-stimulated mice maintained their recovery without further stimulations (Fig. 5). The persistent recovery effect suggests that repeated cLCN stimulations may enhance structural plasticity. Our data on GAP43 supports this, as repeated cLCN stimulations also led to an increase in the axonal growth protein GAP43, and its expression is positively correlated with improved functional outcome (Fig. 4). Future studies will investigate the effects of repeated cLCN stimulations on structural plasticity, including axonal sprouting and dendritic plasticity.

A previous study from Baker *et al*. 2010 showed that electrical stimulation of LCN can yield frequency-dependent changes in cortical excitability^38^. They showed that 30 hz stimulations produced the highest motor evoked potentials; however repeated stimulations can result in variable excitability outcome, and higher frequencies (50–100 hz) produced a trend toward reduced cortical excitability^38^. In our study we used 10 hz to stimulate neurons in the cLCN and observed a clear forelimb movement during the stimulation period (Supplementary Video 1). After one session of stimulation, we observed neuronal activity in the cLCN by pCREB immunostaining (Fig. 2). An important question to investigate is how cLCN stimulations affect cortical excitability, both acutely (after one session of stimulation) and chronically (after 10 days of repeated stimulations). It is possible that cortical excitability may differ between acute and chronic stimulations. This study used the suture stroke model where the ischemic injury can also affect thalamus and hippocampus in addition to striatum and cortex, which makes activation pattern analysis difficult due to the variable stroke locations. Future studies will investigate the activation dynamics during stroke recovery in non-stimulated and cLCN-stimulated mice, using a cortical stroke model where ischemic injury is more consistently localized in the somatosensory cortex.

Most post-stroke brain stimulation studies have focused on cortical stimulation^8^,^39^,^40^. TMS and tDCS can be used non-invasively to manipulate the excitability of the cortical hemisphere^8^,^41^,^42^,^43^. However, these stimulation techniques nonspecifically activate all cell types near the stimulation site, making it difficult to study the underlying mechanisms of stimulation-induced recovery. In this study, our goal was to investigate whether stimulations of LCN neurons alone can drive recovery after stroke. As Thy1 promoter has been shown to be pan-neuronal, we used Thy1-ChR2 mice to stimulate only neurons in the LCN, and thus did not affect other non-neuronal subtypes. Since LCN is a primary source of excitatory output from the cerebellum and we observed visible forelimb movement during stimulation (Supplementary Video 1), it is likely that most of the stimulated cells are excitatory. However, as it has been reported that the LCN contains three neuronal subtypes including glutamatergic, gabaergic and glycinergic^44^,^45^. Thus we do not exclude the possibility that our stimulations may activate other neuronal subtypes that might express ChR2. Future studies will interrogate the effect of stimulating selective neuronal subtypes in the LCN on post-stroke recovery.

Previously we used optogenetics to demonstrate that selective stimulation of neurons in the iM1 promotes recovery after stroke^23^. Although optogenetic stimulation of iM1 enhanced stroke recovery, behavioral testing indicated that the animals had the capacity for further improvement. The motor cortex is a large structure that regulates primarily motor-related functions. An ideal brain target would be a smaller brain region that is easier to target and contains widespread projections that can innervate multiple regions with additional functions, including motor, sensory and non-motor circuits. In this study we highlight the cLCN as a promising brain stimulation target. LCN is anatomically small, yet stimulation of this single site can result in widespread activation of multiple brain regions involved in balance, coordination and visuospatial functions^26^,^28^,^46^. The effect of cLCN stimulation may be more robust than the effects we observed from our previous iM1 stimulation study^23^. This was suggested by our calculations of the slope of speed recovery within each animal in each group (cLCN non-stimulated mice vs cLCN-stimulated mice; iM1 non-stimulated mice vs iM1-stimulated mice) (Supplementary Fig. 3). The average slope of speed recovery over time for the cLCN-stimulated group (cLCN-stimulated vs cLCN non-stimulated mice) appeared steeper than the slope for the iM1-stimulated group (iM1-stimulated vs iM1 non-stimulated mice) (Supplementary Fig. 3), suggesting that cLCN-stimulated mice may recover at a faster rate. Furthermore, the effect of cLCN stimulations is persistent (Fig. 5). Thus cLCN could be an ideal target for deep brain stimulation (DBS) in stroke patients. A recent case study reported that a woman with a cerebellar stroke exhibited improvements in cerebellar ataxia after DBS in the cerebellar LCN, further supporting the feasibility of LCN stimulation for stroke patients^47^. Altogether our results and other cLCN stimulation studies are encouraging and support the potential use of cLCN stimulation in human clinical trials. A single-center study is currently underway at the Cleveland Clinic to evaluate the safety and patient outcomes of electrical stimulation of the LCN for the management of chronic, moderate to severe upper extremity hemiparesis due to ischemic stroke (NCT02835443).

Our data provide strong evidence that cLCN is a promising brain stimulation target for enhancing functional outcome after stroke, and even short term cLCN stimulations can produce persistent effects. A recent study used probabilistic tractography to demonstrate that the dentato-thalamo-cortical tract is positively related to both general motor output and fine motor skills in chronic stroke patients^48^, further highlighting the importance of this circuit in stroke recovery. The effect of cLCN stimulation-enhanced recovery is likely to involve multiple mechanisms, as stimulation of cLCN activates numerous areas, and increasing neural activity would enhance activity-dependent molecules such as cfos and CREB, which are transcription factors that mediate an array of downstream genes^49^,^50^,^51^. Future studies using next generation sequencing will investigate the transcriptome profile of cLCN-stimulated mice, which may provide insight on major pathways underlying stimulation-induced recovery, as well as potential drug target(s) for enhancing stroke recovery.

## Methods

### Animals

For each study, 10–12-week-old Thy1-ChR2-YFP line 18 transgenic male mice were used (B6.Cg-Tg(Thy1-COP4/EYFP)18 Gfng/J). Mice were housed in a 12:12 hour light:dark cycle with food and water provided *ad libitum*. All experimental protocols were conducted in accordance with animal care laws and institutional guidelines, and approved by the Stanford Institutional Animal Care and Use Committee. Because transgenic mice were used, sample size was dependent on breeding efficiency and determined by the number of male transgenic mice available per experiment. Equal numbers were assigned for each group.

### Stereotaxic Surgery

Mice underwent stereotaxic surgery to implant the optical fiber into the cLCN (right side of the brain). Mice were first anesthetized with 5% isoflurane and subsequently maintained with 2–3% isoflurane. Body temperature, heart rate, respiration and mucous membrane color were monitored every 15 minutes. Mice were secured in a digital stereotaxic setup via the ear bars. Artificial tears were used to prevent corneal damage. An incision was made to the top of the scalp and a burr hole was drilled on the right side of the skull without damaging the dura. The optic fiber cannula (200 um) was stereotaxically implanted into the cLCN using these coordinates from the Paxinos and Franklin mouse brain atlas^52^: cLCN coordinate (AP: −5.80 mm, ML: −2.25, DV: 2.05), medial off-target coordinate (AP = −5.80 mm, ML = −1.90 mm, DV = 2.05 mm), lateral off-target coordinate (AP = −5.80 mm, ML = −2.60 mm, DV = 2.05 mm). The optical fiber cannula was secured to the skull using C&B Metabond (Parkell) and dental cement. Wounds were closed with 5–0 sutures and Vetbond tissue glue. Mice were monitored for recovery and returned to their home cages. Mice were randomized and experimenters were blinded. The stereotaxic surgery, stroke surgeries and behavior tests were performed by 3 different individuals.

### Transient Middle Cerebral Artery Occlusion

Mice were anesthetized with 5% isoflurane then maintained on 2–3% isoflurane and physiological parameters were monitored as mentioned above. Artificial tears were used to prevent corneal damage. An intraluminal suture (70SPRePK5–2045, 0.20 mm diameter, 4–5 mm length, Doccol Corporation) was inserted into the left internal carotid artery to block the blood flow to the middle cerebral artery, which will produce an infarct on the left side of the brain. The suture was left in place for 30 minutes and removed to allow reperfusion. Wounds were closed with suturing and tissue glue, and mice were administered buprenorphine (0.05–0.1 mg/kg) and 0.9% saline (subcutaneously). Mice were monitored for recovery and returned to their home cages. All mice were injected with 0.5 ml of 0.9% saline subcutaneously every day for 7 days after stroke to help prevent dehydration.

### Stimulation Paradigm

All experimental groups underwent identical behavior handling/training, surgical procedures (stroke and fiber implant in cLCN) and environmental exposures, with the exception that control non-stimulated stroke mice did not receive laser pulses for stimulations. Each mouse was placed in an empty mouse cage after a laser cable was connected to its fiber cannula. During stimulations the animal was allowed to freely move in the empty mouse cage. In the cLCN stimulated group, each mouse received one session of stimulation which consisted of three 1-minute stimulations separated by 3-minute rest intervals. The cLCN short stimulated group received stimulations daily from post-stroke Days 5–14 (6 days/wk) and the cLCN long stimulated group received stimulations daily from post-stroke Days 5–28 (6 days/wk). A 473 nm blue laser (OEM Laser Systems, Salt Lake City, UT) was controlled by the Agilent function generator (AGT33210A) and mice were stimulated with a laser set to 10 Hz, 20 msec light pulses with a power range of 0.2–0.4 mW as measured by a power meter (Thor labs, Newton, New Jersey). We used the minimal laser power necessary to elicit movements in the affected forelimb during stimulations. Each animal was stimulated at a slightly different power level based on the observation of forelimb movements that day. Stimulations were performed in the morning between 10–12 pm and behavior tests were performed in the afternoon between 2–5 pm, thus allowing for ~4 hours between stimulations and behavior testing. One session of stimulation was given to all mice (pre-stroke test stimulation) after the pre-stroke baseline (the day before stroke) in order to evaluate a positive forelimb movement response during laser stimulation. Lack of response would indicate misplacement of fiber implant or absence of ChR2 gene expression to achieve activation. Animals that did not exhibit visible forelimb movements during stimulations were excluded in the study.

### Rotating beam test

The rotating beam test is a motor/sensory test to detect neurological deficits after stroke. We measured the distance and speed traveled of mice placed on a rotating white fiberglass beam (length 120 cm, diameter 13 mm, distances marked every cm). The rotating beam (3 rpm) is situated at 60 cm above the floor with bubble cushions covering the floor to reduce the impact from a fall. Three trials were performed for each mouse and the two highest performance values for distance and speed were averaged for data analysis. Behavior testing and optogenetic stimulations were performed by different double-blinded experimenters.

Mice were trained on the rotating beam test 3–4 times before the baseline data were recorded. Two baselines were collected: the pre-implant baseline was the day before implantation of the optical fiber and the pre-stroke baseline was the day before stroke surgery. After stroke, the rotating beam test was performed on post-stroke Days 4, 7, 10 and 14 for the short stimulation study. For the 1-month study investigating the persistence of the stimulation effects, we performed behavior tests on post-stroke Days 4, 7, 14, 21 and 28. Exclusion criteria: 1) mice exhibiting behavioral deficits after implantation, 2) mice without behavioral deficits at Day 4 after stroke, and 3) mice without cortical infarcts (validated with histology after sacrifice). Mice included in the study were randomly assigned into two groups (no stim and stim) using an in-house software program that randomly balanced the groups based on two variables: behavior performance at the 1) pre-stroke baseline and 2) post-stroke Day 4.

### Immunofluorescence Staining

At Day 15 (short term study) or Day 29 (long term study) after stroke, mice were sacrificed and perfused with cold PBS and 3% paraformaldehyde. Brains were cryoprotected overnight in a 20% sucrose/3%PFA solution. After the brains sank to the bottom they were frozen on dry ice and stored at −80 °C until sectioning. The brains were sectioned on a cryostat at 30 μm and kept at −20 °C in an antifreeze solution (30% ethylene glycol, 30% glycerol in PBS). Sections were washed in 0.3% PBS-triton, transferred to a blocking solution (5% species serum, 1% bovine serum albumin in 0.3% PBS-triton) for 2 hours, and incubated overnight at 4 °C in a solution of primary antibody (pCREB, Cell Signaling #9198, dilution 1:200; CD68, Abcam ab53444, dilution 1:200; GFAP, Abcam ab4674, dilution 1:500) diluted in blocking solution. The next day, sections were washed in 0.3% PBS-triton and incubated for 2 hours at room temperature with 1:500 secondary antibody (either Alexa fluor 488, 555, 594 or 647) diluted in blocking solution. DAPI (1:2000) was introduced during the last 5 minutes of the secondary antibody incubation. Sections were then washed in PBS, mounted in Fluoromount (Sigma-Aldrich, F4680) and cover-slipped. Procedures for the double immunofluorescence staining is identical to the single immunostaining as described above, with the exception of: 1) Two primary antibodies (made from different species) were added together during the primary antibody incubation period; 2) Two secondary antibodies (target different species) were added together during the secondary antibody incubation period.

### Infarct quantitation and imaging

All mice processed for immunostaining were stained with CD68 to verify their infarct size and location. Some mice were also stained with GFAP as an additional marker to visualize infarct. Images were acquired and stitched using Confocal LSM800. Infarct area, ipsilesional hemisphere and contralesional hemisphere were outlined and these areas were calculated using NIH Image J. Percentage of infarct size = 100 × [contralesional hemisphere area − (total ipsilesional hemisphere area − infarct area)]/total contralesional hemisphere area. Average percent infarct size was measured and calculated from two levels: the striatum level and the hippocampus level. All of the whole brain immunostained images were taken from the Confocal LSM800. The pCREB images were taken from the Zeiss Axio-imager. All images were processed into figures using Adobe Photoshop.

### Photothrombotic lesion model

The photothrombotic model can lesion a target area through photo-activation of an injected light-sensitive dye (Rose Bengal)^36^. Following light illumination, Rose Bengal is activated and leads to damage in the cell membranes of endothelial cells, resulting in interruption of local blood flow and lesioning of the target area. The photosensitive dye Rose Bengal (10 mg/mL, Sigma #330000) was made with 0.9% sterile saline. Mice with fiber implants were injected with Rose Bengal intraperitoneally (8 ul/g). Five minutes after Rose Bengal injection, the mice were exposed to a green laser (530 nm) through the fiber cannula for 10 minutes (laser power at 2 mW). Mice were returned to their cage after the photothrombotic model. After 24–48 hours, mice were sacrificed and brains were stained with GFAP and MAP2 for histological verification of the implant location and lesion area.

### Western blot analysis

At Day 15 after stroke, some mice were sacrificed and perfused with cold PBS. Brain regions of interest were dissected on ice and quickly frozen to −80 °C. Proteins were extracted using the NE-PER nuclear and cytoplasmic extraction kit (ThermoFisher). Protein concentrations were measured using the Bradford Assay and all protein samples were diluted to equal concentrations (1 ug/ul for cytoplasmic samples). Proteins were separated by SDS gel electrophoresis performed on a 4–20% TGX Ready Gel (Bio-Rad) at 200 V, then proteins were transferred to a PVDF membrane using the Trans-blot Turbo system (Bio-Rad) and blocked in 5% milk to prevent nonspecific binding. Membranes were then incubated in primary antibodies (GAP43, Cell Signaling #D9C8, dilution 1:1000) diluted in 5% milk overnight at 4 °C. The next day the membranes were incubated in secondary antibody (Anti-rabbit-HRP) at room temperature for 1 hour. Membranes were also blotted with the housekeeping gene GAPDH (Cell Signaling #3683, dilution 1:5000) for 1 hour at room temp. The Amersham ECL Prime Western Blotting Detection Reagent (RPN2232) was used to develop the membranes and protein expression signals were captured using the Bio-Rad Chemi-Doc MP Imager. NIH ImageJ was used to calculate relative optical density as previously described^23^. After quantitation of relative optical density, the level of GAP43 protein was normalized using GAPDH.

### Statistics

All statistics were performed using Prism 5.0. Behavior analyses were statistically analyzed with two-way ANOVA followed by Bonferroni’s post hoc test. The western data and infarct data were statistically analyzed by Student’s t-test, two-tailed. Please refer to figure legends for details on the statistics.

## Additional Information

**How to cite this article**: Shah, A. M. *et al*. Optogenetic neuronal stimulation of the lateral cerebellar nucleus promotes persistent functional recovery after stroke. *Sci. Rep.* **7**, 46612; doi: 10.1038/srep46612 (2017).

**Publisher's note:** Springer Nature remains neutral with regard to jurisdictional claims in published maps and institutional affiliations.

## Supplementary Material

Supplementary Information

Supplementary Video 1

Supplementary Video 2

Supplementary Video 3

## Figures and Tables

**Figure 1:**
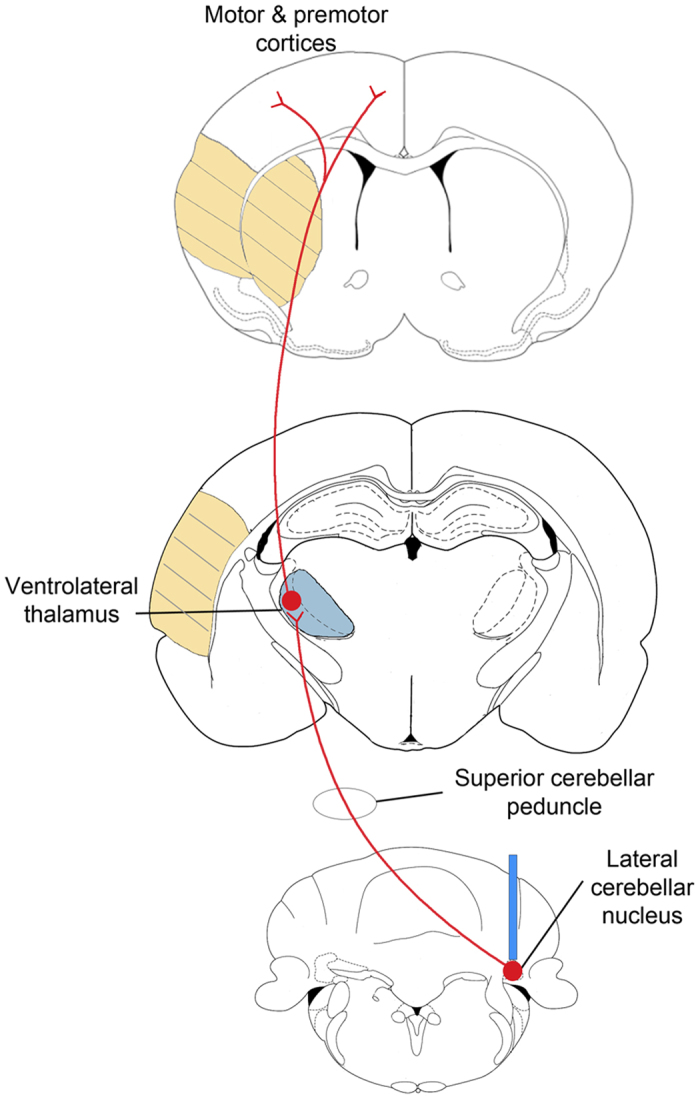
Stimulation target: Dentato-thalamo-cortical pathway. Diagram depicting the projections from the lateral cerebellar nucleus (bottom) to ventrolateral thalamus (middle) to cortex (top). At the lateral cerebellar nucleus (LCN) level, the blue bar in the LCN indicates the location of fiber cannula implant (contralesional LCN). This tract, originating from neurons in the LCN, projects through the ipsilateral superior cerebellar peduncle. After decussating in the midbrain tegmentum, the axons synapse in the contralesional ventrolateral thalamus (blue) from which second order neurons send projections to multiple cortical regions including the premotor, primary motor, prefrontal and posterior parietal areas. The region of the yellow hatch marks represents the infarct areas (striatum and cortex) produced by transient middle cerebral artery occlusion.

## References

[b1] Carmichael S. T. Plasticity of Cortical Projections after Stroke. Neurosci. 9, 64–75 (2003). 10.1177/1073858402239592 12580341

[b2] Murphy T. H. & Corbett D. Plasticity during stroke recovery: from synapse to behaviour. Nat. Rev. Neurosci. 10, 861–72 (2009). 19888284 10.1038/nrn2735

[b3] Grefkes C. & Ward N. S. Cortical reorganization after stroke: how much and how functional? Neuroscientist 20, 56–70 (2014). 23774218 10.1177/1073858413491147

[b4] Silasi G. & Murphy T. H. Stroke and the Connectome: How Connectivity Guides Therapeutic Intervention. Neuron 83, 1354–1368 (2014). 25233317 10.1016/j.neuron.2014.08.052

[b5] Clarkson A. N., Huang B. S., Macisaac S. E., Mody I. & Carmichael S. T. Reducing excessive GABA-mediated tonic inhibition promotes functional recovery after stroke. Nature 468, 305–9 (2010). 21048709 10.1038/nature09511 PMC3058798

[b6] Hiu T. . Enhanced phasic GABA inhibition during the repair phase of stroke: A novel therapeutic target. Brain 139, 468–480 (2015). 26685158 10.1093/brain/awv360 PMC4805083

[b7] Andres R. H. . Human neural stem cells enhance structural plasticity and axonal transport in the ischaemic brain. Brain 134, 1777–89 (2011). 21616972 10.1093/brain/awr094 PMC3102243

[b8] Webster B. R., Celnik P. A. & Cohen L. G. Noninvasive Brain Stimulation in Stroke Rehabilitation. NeuroRx 3, 474–481 (2006). 17012061 10.1016/j.nurx.2006.07.008 PMC3593409

[b9] Plow E. B., Carey J. R., Nudo R. J. & Pascual-Leone A. Invasive cortical stimulation to promote recovery of function after stroke: a critical appraisal. Stroke. 40, 1926–31 (2009). 19359643 10.1161/STROKEAHA.108.540823 PMC3232009

[b10] Taub E. & Morris D. M. Constraint-induced movement therapy to enhance recovery after stroke. Curr. Atheroscler. Rep. 3, 279–86 (2001). 11389792 10.1007/s11883-001-0020-0

[b11] Brown J. a., Lutsep H. L., Weinand M. & Cramer S. C. Motor cortex stimulation for the enhancement of recovery from stroke: a prospective, multicenter safety study. Neurosurgery 58, 464–73 (2006). 16528186 10.1227/01.NEU.0000197100.63931.04

[b12] Paquette C., Sidel M., Radinska B. A., Soucy J.-P. & Thiel A. Bilateral transcranial direct current stimulation modulates activation-induced regional blood flow changes during voluntary movement. J. Cereb. blood flow Metab. 31, 2086–95 (2011). 21559029 10.1038/jcbfm.2011.72 PMC3208154

[b13] Takeuchi N. & Izumi S.-I. Noninvasive brain stimulation for motor recovery after stroke: mechanisms and future views. Stroke Res. Treat. 2012, 584727 (2012). 23050198 10.1155/2012/584727 PMC3463193

[b14] Bashir S., Mizrahi I., Weaver K., Fregni F. & Pascual-Leone A. Assessment and modulation of neural plasticity in rehabilitation with transcranial magnetic stimulation. PM R 2, S253–68 (2010). 21172687 10.1016/j.pmrj.2010.10.015 PMC3951769

[b15] Fregni F. & Pascual-Leone A. Technology insight: noninvasive brain stimulation in neurology-perspectives on the therapeutic potential of rTMS and tDCS. Nat. Clin. Pract. Neurol. 3, 383–93 (2007). 17611487 10.1038/ncpneuro0530

[b16] Fenoy A. J., Goetz L., Chabardès S. & Xia Y. Deep Brain Stimulation: Are Astrocytes a Key Driver Behind the Scene? CNS Neurosci. Ther. 20, 191–201 (2014). 24456263 10.1111/cns.12223 PMC3969941

[b17] Blomstedt P. & Hariz M. I. Are complications less common in deep brain stimulation than in ablative procedures for movement disorders? Stereotact. Funct. Neurosurg. 84, 72–81 (2006). 16790989 10.1159/000094035

[b18] Alhourani A. . Network effects of deep brain stimulation. J. Neurophysiol. 114, 2105–17 (2015). 26269552 10.1152/jn.00275.2015 PMC4595613

[b19] Yizhar O., Fenno L. E., Davidson T. J., Mogri M. & Deisseroth K. Optogenetics in neural systems. Neuron 71, 9–34 (2011). 21745635 10.1016/j.neuron.2011.06.004

[b20] Fenno L., Yizhar O. & Deisseroth K. The development and application of optogenetics. Annu. Rev. Neurosci. 34, 389–412 (2011). 21692661 10.1146/annurev-neuro-061010-113817 PMC6699620

[b21] Cheng M. Y., Wang E. H. & Steinberg G. K. Optogenetic Approaches to Study Stroke Recovery. 5–6, doi: 10.1038/nrn2735.(3) (2014). 25259689

[b22] Cheng M. Y., Aswendt M. & Steinberg G. K. Optogenetic Approaches to Target Specific Neural Circuits in Post-stroke Recovery. Neurotherapeutics 13, 325–40 (2016). 26701667 10.1007/s13311-015-0411-5 PMC4824024

[b23] Cheng M. Y. . Optogenetic neuronal stimulation promotes functional recovery after stroke. Proc. Natl. Acad. Sci. 1–6, doi: 10.1073/pnas.1404109111 (2014). PMC4156770 25136109

[b24] Strick P. L., Dum R. P. & Fiez J. A. Cerebellum and nonmotor function. Annu. Rev. Neurosci. 32, 413–34 (2009). 19555291 10.1146/annurev.neuro.31.060407.125606

[b25] Purves D. . Projections from the Cerebellum. (2001).

[b26] Dum R. P., Li C. & Strick P. L. Motor and nonmotor domains in the monkey dentate. Ann. N. Y. Acad. Sci. 978, 289–301 (2002). 12582061 10.1111/j.1749-6632.2002.tb07575.x

[b27] Dum R. P. & Strick P. L. An unfolded map of the cerebellar dentate nucleus and its projections to the cerebral cortex. J. Neurophysiol. 89, 634–9 (2003). 12522208 10.1152/jn.00626.2002

[b28] Küper M. . Evidence for a motor and a non-motor domain in the human dentate nucleus–an fMRI study. Neuroimage 54, 2612–22 (2011). 21081171 10.1016/j.neuroimage.2010.11.028

[b29] Liepert J. . Motor cortex excitability after cerebellar infarction. Stroke. 35, 2484–8 (2004). 15375297 10.1161/01.STR.0000143152.45801.ca

[b30] Machado A. G., Baker K. B., Schuster D., Butler R. S. & Rezai A. Chronic electrical stimulation of the contralesional lateral cerebellar nucleus enhances recovery of motor function after cerebral ischemia in rats. Brain Res. 1280, 107–16 (2009). 19445910 10.1016/j.brainres.2009.05.007 PMC2709491

[b31] Cooperrider J. . Chronic Deep Cerebellar Stimulation Promotes Long-Term Potentiation, Microstructural Plasticity, and Reorganization of Perilesional Cortical Representation in a Rodent Model. J. Neurosci. 34, 9040–9050 (2014). 24990924 10.1523/JNEUROSCI.0953-14.2014 PMC4078081

[b32] Stroemer R. P., Kent T. A. & Hulsebosch C. E. Neocortical Neural Sprouting, Synaptogenesis, and Behavioral Recovery After Neocortical Infarction in Rats. Stroke 26, 2135–2144 (1995). 7482662 10.1161/01.str.26.11.2135

[b33] Carmichael S. T. . Growth-associated gene expression after stroke: evidence for a growth-promoting region in peri-infarct cortex. Exp. Neurol. 193, 291–311 (2005). 15869933 10.1016/j.expneurol.2005.01.004

[b34] Aigner L. . Overexpression of the neural growth-associated protein GAP-43 induces nerve sprouting in the adult nervous system of transgenic mice. Cell 83, 269–278 (1995). 7585944 10.1016/0092-8674(95)90168-x

[b35] Schoenenberger P., Schärer Y.-P. Z. & Oertner T. G. Channelrhodopsin as a tool to investigate synaptic transmission and plasticity. Exp. Physiol. 96, 34–39 (2011). 20562296 10.1113/expphysiol.2009.051219

[b36] Labat-gest V. & Tomasi S. Photothrombotic Ischemia: A Minimally Invasive and Reproducible Photochemical Cortical Lesion Model for Mouse Stroke Studies. J. Vis. Exp., doi: 10.3791/50370 (2013) PMC3727176 23770844

[b37] Gradinaru V. . Targeting and readout strategies for fast optical neural control *in vitro* and *in vivo*. J. Neurosci. 27, 14231–8 (2007). 18160630 10.1523/JNEUROSCI.3578-07.2007 PMC6673457

[b38] Baker K. B., Schuster D., Cooperrider J. & Machado A. G. Deep brain stimulation of the lateral cerebellar nucleus produces frequency-specific alterations in motor evoked potentials in the rat *in vivo*. Exp. Neurol. 226, 259–64 (2010). 20816822 10.1016/j.expneurol.2010.08.019 PMC2971530

[b39] Nowak D. A. . Effects of low-frequency repetitive transcranial magnetic stimulation of the contralesional primary motor cortex on movement kinematics and neural activity in subcortical stroke. Arch. Neurol. 65, 741–7 (2008). 18541794 10.1001/archneur.65.6.741

[b40] Adkins D. L., Hsu J. E. & Jones T. A. Motor cortical stimulation promotes synaptic plasticity and behavioral improvements following sensorimotor cortex lesions. Exp. Neurol. 212, 14–28 (2008). 18448100 10.1016/j.expneurol.2008.01.031 PMC3018150

[b41] Liew S.-L., Santarnecchi E., Buch E. R. & Cohen L. G. Non-invasive brain stimulation in neurorehabilitation: local and distant effects for motor recovery. Front. Hum. Neurosci. 8, 378 (2014). 25018714 10.3389/fnhum.2014.00378 PMC4072967

[b42] Feng W. W., Bowden M. G. & Kautz S. Review of transcranial direct current stimulation in poststroke recovery. Top. Stroke Rehabil. 20, 68–77 (2013). 23340073 10.1310/tsr2001-68

[b43] Schlaug G., Renga V. & Nair D. Transcranial direct current stimulation in stroke recovery. Arch. Neurol. 65, 1571–1576 (2008). 19064743 10.1001/archneur.65.12.1571 PMC2779259

[b44] Uusisaari M. & Knöpfel T. GlyT2+ neurons in the lateral cerebellar nucleus. Cerebellum 9, 42–55 (2010). 19826891 10.1007/s12311-009-0137-1 PMC2840673

[b45] Uusisaari M. & Knöpfel T. Functional classification of neurons in the mouse lateral cerebellar nuclei. Cerebellum 10, 637–46 (2011). 21116763 10.1007/s12311-010-0240-3 PMC3215887

[b46] Dum R. P. & Strick P. L. An unfolded map of the cerebellar dentate nucleus and its projections to the cerebral cortex. J. Neurophysiol. 89, 634–639 (2003). 12522208 10.1152/jn.00626.2002

[b47] Teixeira M. J. . Deep brain stimulation of the dentate nucleus improves cerebellar ataxia after cerebellar stroke. Neurology 85, 2075–6 (2015). 26644050 10.1212/WNL.0000000000002204

[b48] Schulz R. . Cortico-Cerebellar Structural Connectivity Is Related to Residual Motor Output in Chronic Stroke. Cereb. Cortex bhv251 (2015). 10.1093/cercor/bhv251 26508336

[b49] Kitagawa K. CREB and cAMP response element-mediated gene expression in the ischemic brain. FEBS J. 274, 3210–3217 (2007). 17565598 10.1111/j.1742-4658.2007.05890.x

[b50] Impey S. . Defining the CREB RegulonA Genome-Wide Analysis of Transcription Factor Regulatory Regions. Cell 119, 1041–1054 (2004). 15620361 10.1016/j.cell.2004.10.032

[b51] Johansson I.-M. . Early and delayed induction of immediate early gene expression in a novel focal cerebral ischemia model in the rat. Eur. J. Neurosci. 12, 3615–3625 (2000). 11029632 10.1046/j.1460-9568.2000.00252.x

[b52] Paxinos G. & Franklin K. B. J. Paxinos and Franklin’s the Mouse Brain in Stereotaxic Coordinates. São Paulo. Academic Press (2012).

