# Supplementary material for: Optogenetic neuronal stimulation of the lateral cerebellar nucleus promotes persistent functional recovery after stroke
Source: Sci Rep. 2017 Jun 1;7:46612. doi: 10.1038/srep46612 (PMC5451884; doi:10.1038/srep46612)
Supplement: Supplementary Information [file srep46612-s1.pdf]

Supplementary Information

Optogenetic neuronal stimulation of the lateral cerebellar nucleus promotes persistent functional recovery after stroke

Aatman M. Shah[1,2], Shunsuke Ishizaka[1,2], Michelle Y. Cheng[1,2*], Eric H. Wang[1], Alex R. Bautista[1], Sabrina Levy[1], Daniel Smerin[1], Guohua Sun[1], Gary K. Steinberg[1*]

[1]Department of Neurosurgery, Stanford, CA 94305

[2]Co-first authors

*Corresponding authors: Gary K. Steinberg (gsteinberg@stanford.edu) and Michelle Y. Cheng (mycheng@stanford.edu)

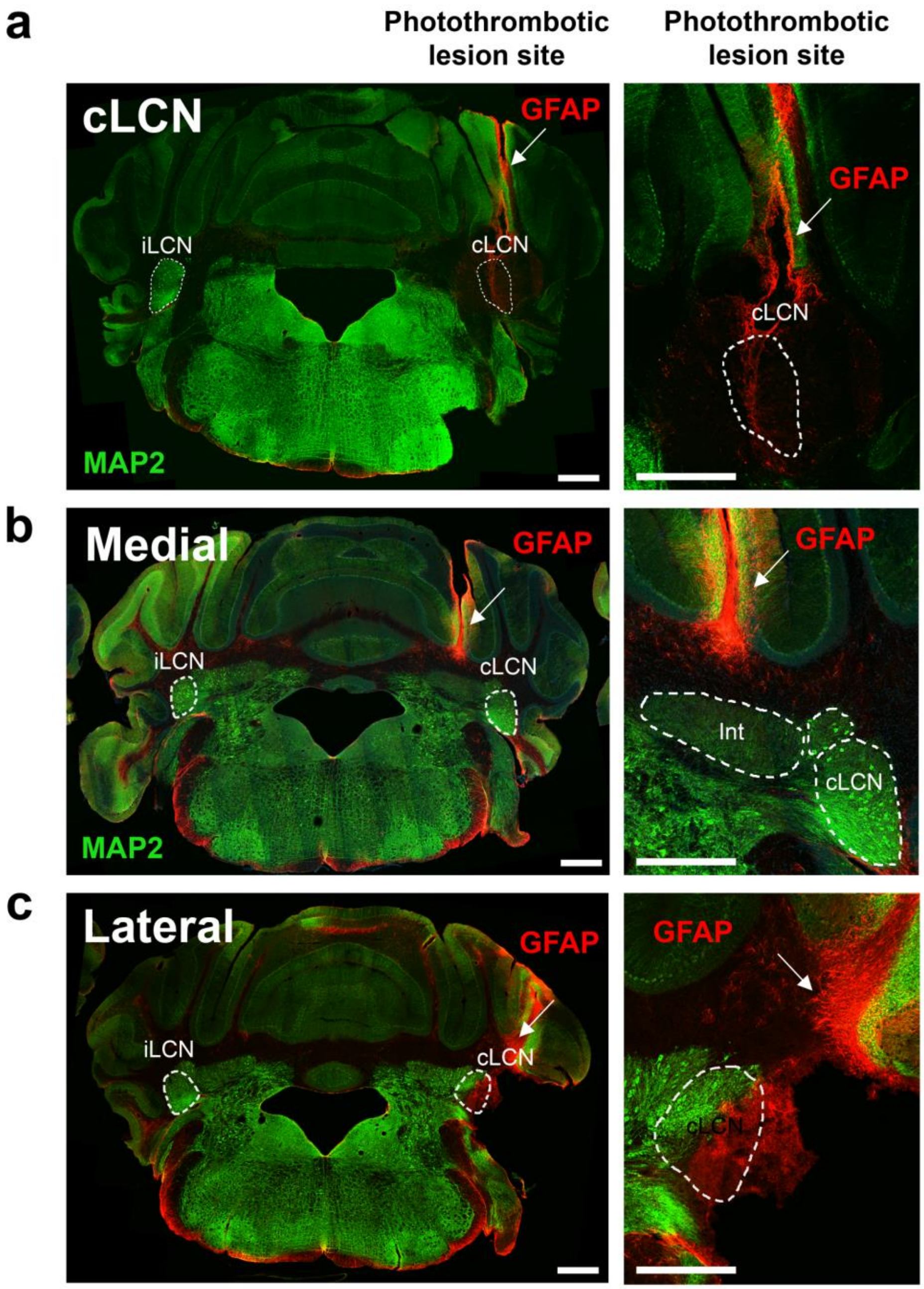

Supplementary Figure 1: Validation of cLCN implant location via photothrombotic lesion method. Representative images of cLCN implant location and lesion location in the (**a**) cLCN-implant mouse, (**b**) medial-implant mouse and (**c**) lateral-implant. Mice were double-immunostained with GFAP (red) to visual the implant track location and MAP2 (green) to visualize neuronal structure. Note that cLCN-implanted mouse exhibit successful lesion of the cLCN after photothrombotic method (**a**). Medial-implant did not affect cLCN (**b**), while lateral-implant partially lesioned the cLCN (**c**). Scale bar = 500um.

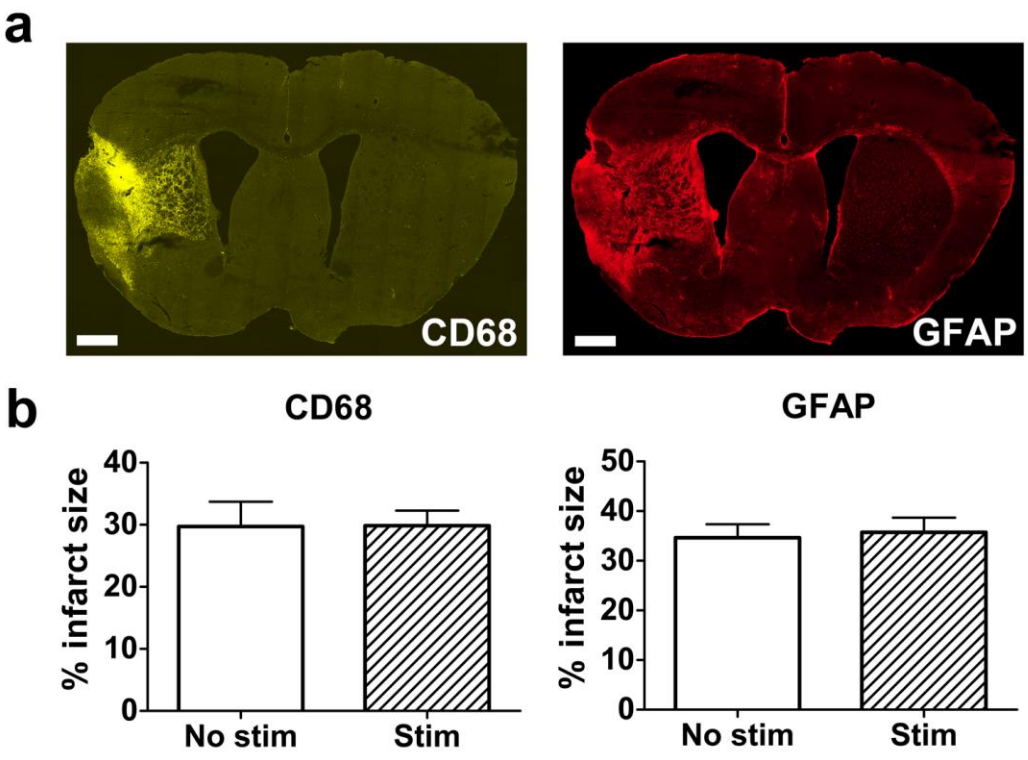

Supplementary Figure 2: cLCN stimulation did not affect infarct size. (**a**) Representative images of CD68 and GFAP immunostaining illustrating infarct location. Scale bar = 1000um. (**b**) Quantification of percent infarct size in no stim and stim stroke mice (left: CD68, right: GFAP). cLCN stimulation did not affect infarct size in our study. n=6 for no stim group and n=7 for stim group. Data are expressed as mean ± s.e.m.

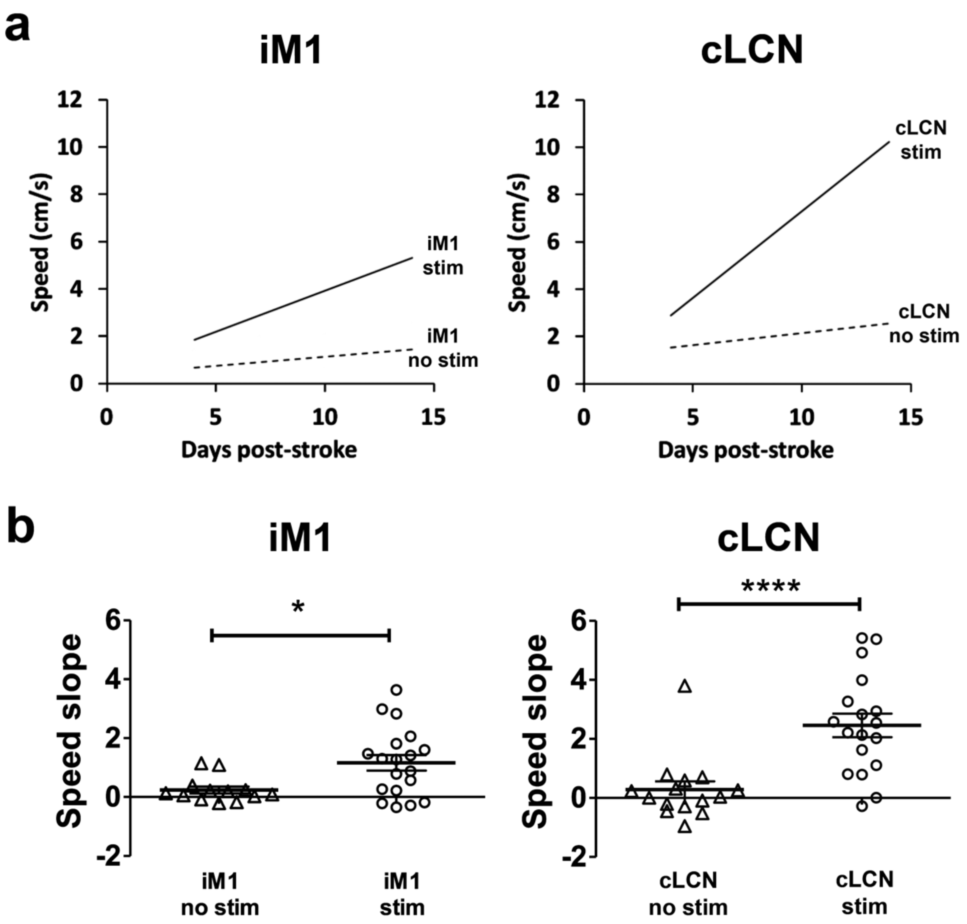

Supplementary Figure 3: Rate of recovery in cLCN stimulations and iM1 stimulations. We compared the rate of recovery between cLCN and iM1 stimulations. The rate of recovery was represented by the slopes of the linear regression line from each group, which was calculated from their average speed performed over time. iM1 behavior data was taken from a previous group of iM1 stimulated mice and we calculated their rate of recovery in order to compare to cLCN stimulated mice in this study (iM1 slope data has not been previously reported). (**a**) Graph illustrates the slope of recovery in iM1 group (Left: iM1 no stim vs iM1 stim) and cLCN group (Right: cLCN no stim vs cLCN stim). Note that the fitted trend lines provide visualization of the slopes for each group, which suggest a faster recovery in the cLCN stimulated group. (**b**) Graph depicts comparison of individual mice speed slopes for the iM1 and cLCN groups. iM1-stimulated mice has a significantly faster slope the iM1 no stim mice. n=13 for iM1 no stim, n=19 for iM1 stim, **P*<0.05, Student's t-test, two-tailed. cLCN-stimulated mice has a significantly faster slope than cLCN no stim mice. n=15 for cLCN no stim, n=18 for cLCN stim, *****P*<0.0001,Student's t-test, two-tailed. Data are expressed as mean ± s.e.m.

Supplementary Video 1: Visualization of forelimb movements in cLCN implant mouse. The video shows a cLCN-implant mouse during stimulation OFF and ON periods. Note that significant forelimb movement was induced (on the same side of implant) during stimulation ON period. This visual validation of cLCN implant location is one of our criteria for inclusion in the study. Mice that lack visual forelimb movements during stimulation were excluded.

Supplementary Video 2: Visualization of forelimb movements in the off target medial-implant mouse. The video shows the off target medial-implant mouse during stimulation OFF and ON periods. Note that no forelimb movement was present during stimulation ON period.

Supplementary Video 3: Visualization of forelimb movements in the off target lateral-implant mouse. The video shows off target lateral-implant mouse during stimulation OFF and ON periods. Note that no forelimb movement was present during stimulation ON period.
